# Comorbidities and Burden of COPD: A Population Based Case-Control Study

**DOI:** 10.1371/journal.pone.0063285

**Published:** 2013-05-17

**Authors:** Florent Baty, Paul Martin Putora, Bruno Isenring, Torsten Blum, Martin Brutsche

**Affiliations:** 1 Division of Pulmonary Medicine, Cantonal Hospital, St. Gallen, Switzerland; 2 Department of Radiation Oncology, Cantonal Hospital, St. Gallen, Switzerland; 3 Clinic of Pulmonary Medicine, University Hospital Zurich, Zurich, Switzerland; 4 Department of Pulmonary Medicine, Lung Clinic Heckeshorn, Berlin, Germany; Pulmonary Research Institute at LungClinic Grosshansdorf, United States of America

## Abstract

COPD is associated with a relevant burden of disease and a high mortality worldwide. Only recently, the importance of comorbidities of COPD has been recognized. Studies postulated an association with inflammatory conditions potentially sharing pathogenic pathways and worsening overall prognosis. More evidence is required to estimate the role of comorbidities of COPD. Our aim was to investigate the prevalence and clustering of comorbidities associated with COPD, and to estimate their impact on clinically relevant outcomes. In this population-based case-control study, a nation-wide database provided by the Swiss Federal Office for Statistics enclosing every hospital entry covering the years 2002–2010 (*n* = 12′888′075) was analyzed using MySQL and R statistical software. Statistical methods included non-parametric hypothesis testing by means of Fisher’s exact test and Wilcoxon rank sum test, as well as linear models with generalized estimating equation to account for intra-patient variability. Exploratory multivariate approaches were also used for the identification of clusters of comorbidities in COPD patients. In 2.6% (6.3% in patients aged >70 years) of all hospitalization cases an active diagnosis of COPD was recorded. In 21% of these cases, COPD was the main reason for hospitalization. Patients with a diagnosis of COPD had more comorbidities (7 [IQR 4–9] vs. 3 [IQR 1–6]; 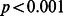), were more frequently rehospitalized (annual hospitalization rate 0.33 [IQR 0.20–0.67] vs. 0.25 [IQR 0.14–0.43]/year; 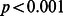), had a longer hospital stay (9 [IQR 4–15] vs. 5 [IQR 2–11] days; 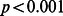), and had higher in-hospital mortality (5.9% [95% CI 5.8%–5.9%] vs. 3.4% [95% CI 3.3%–3.5%]; 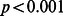) compared to matched controls. A set of comorbidities was associated with worse outcome. We could identify COPD-related clusters of COPD-comorbidities.

## Introduction

Chronic obstructive pulmonary disease (COPD) is an umbrella term including various and sometimes disregarded disorders or conditions [1], namely bronchitis and emphysema with its subsets and a certain overlap with asthmatic disease [2].

The definition of COPD was recently reformulated by the Global Initiative for Chronic Obstructive Lung Disease (GOLD). It states that chronic obstructive pulmonary disease (COPD) is a common preventable and treatable disease, which is characterized by persistent airflow limitation that is usually progressive and associated with an abnormal inflammatory response of the lung to noxious particles or gases. Exacerbations and comorbidities contribute to the overall severity in individual patients [3].

Beside pulmonary limitations, frequently aggravated by exacerbations, the clinical course as well as the prognosis of COPD patients seems to be significantly influenced by comorbidities, which led to the concept of “COPD comorbidome” [4]. Previous review articles have systematically summarized evidence demonstrating relevant associations between COPD and the following diseases [4]–[10]:

- **Cardiovascular diseases**: ischemic heart disease, cerebrovascular disease, peripheral artery disease, left failure, right heart failure and pulmonary hypertension, arrhythmias (atrial fibrillation and flutter), arterial hypertension
- **Respiratory tract diseases**: obstructive sleep apnea, pneumonia, lung fibrosis
- **Metabolic diseases**: metabolic syndrome, type II diabetes mellitus, dyslipidemia
- **Haematological diseases/coagulopathies**: secondary polycythemia, anaemia, venous thrombosis and pulmonary embolism
- **Musculoskeletal diseases**: muscle dysfunction, muscle wasting, osteoporosis
- **Gastro-intestinal diseases**: Gastro-oesophageal reflux disease, peptic ulcer disease, liver cirrhosis
- **Renal diseases**: renal dysfunction
- **Psychiatric diseases**: depression, anxiety
- **Neoplasias**: lung, esophageal, pancreatic, breast, and all other cancers

Explicit associations to COPD have not been defined for all of these comorbidities yet. Aging with generally increasing incidences of comorbidities might be considered as a potential confounder. In the same way, common causative factors (*e.g.* exposure to tobacco smoke) could explain co-existing, but independent diseases aside from COPD. However, there is a significantly higher prevalence for the majority of the named comorbidities in COPD patients compared to individuals without COPD when adjusted for age and risk factors [5], [7], [8]. Some comorbidities are evoked or at least deteriorated by means of specific anatomico-mechanical alterations within the course of COPD. For example, hyperinflation can impair ventricular filling and consequently worsen heart failure [11]. In addition, some pathophysiological sequelae of COPD are accounted for a disproportional impairment of comorbid diseases. Physical inactivity is for instance a common feature and negative predictor in COPD as well as many of the listed comorbid diseases [12], [13].

Moreover, current evidence sustainably supports the hypothesis that chronic systemic inflammation constitutes the key link between COPD and some of its related comorbidities [14], [15]. Nevertheless, different origins of the systemic inflammation have been postulated. Some authors favor a spill-over of inflammatory mediators originating from chronic bronchial and pulmonary inflammation in COPD [7], [16]. Alternatively COPD has been interpreted to be one part of a chronic systemic inflammatory syndrome among other inflammatory entities (*i.e.* atherosclerotic diseases) encompassing particular local manifestations of one superordinate trigger, be they innate or acquired, might be a crucial step in the pathogenesis of these chronic systemic inflammatory syndromes including the development of COPD [17], [18].

Despite the enhanced appreciation of comorbidities in COPD, there is still considerable ambiguity on their respective prevalence, as well as their specific impact in COPD patients in the context of a general population-based perspective as most related evidence has been obtained by studies with highly selected patient populations.

The aim of this study is to clarify prevalence and prognostic relevance of specific COPD comorbidities in the entire and unselected population of Switzerland utilizing the nation-wide in-patient database of the Swiss Federal Office for Statistics.

## Results

### Characteristics of COPD Hospitalizations in Switzerland

**Figure 1:**
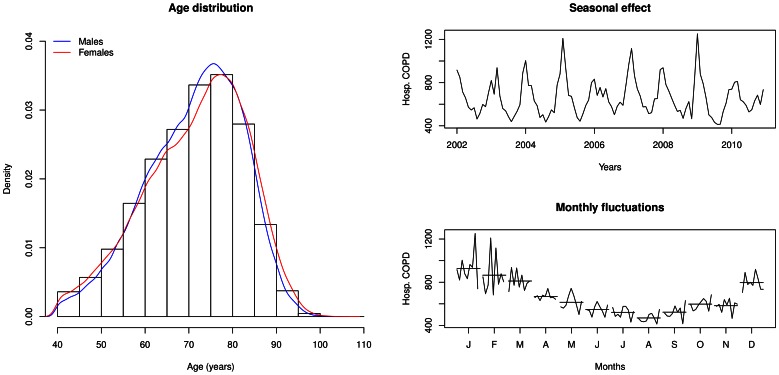
Age, gender and seasonal distribution of COPD hospitalizations. The left panel displays the age distribution stratified by gender of patients with COPD diagnosis. The right panels show the seasonal effect in the hospitalization of acute COPD patients (COPD as main diagnosis), over the 2002–2010 period (upper right panel), and further summarized over monthly fluctuations (lower right panel).

Because COPD diagnosis is very uncommon under the age of 40 years, patients 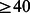 years of age were excluded from the analyses. The number of hospitalizations with a diagnosis of COPD between 2002 and 2010 was 340′948 corresponding to 160′317 unique patients (Table 1). Thus 2.6% (340′948/12′888′075) of all hospitalizations in Switzerland were related to COPD. This percentage increases from 1.0% in younger patients (40–50 year-old) up to 6.3% in elderly patients (more than 70 year-old). In 21% of cases (71′191/340′948), COPD was the main diagnosis, mostly due to acute exacerbation. Sixty four percent were male, and the median age was 73 years (IQR: 64–80). As to be expected, there were seasonal variations over the observation period with peaks during the winter/influenza season (Figure 1).

**Table 1:** Characteristics of patients with COPD compared to age- and sex-matched patients without diagnosis of COPD (controls).

	COPD	Controls	*p*-value
	(*n* = 340′948)	(*n* = 340′948)	
Median age in years [IQR]	73 [64–80]	*	–
Sex-ratio (male:female)	64%	*	–
Number of unique patients	160′317	298′359	–
Median hospitalization rate per year [IQR]	0.33 [0.20–0.67]	0.25 [0.14–0.43]	<0.001
Median time between 2 hospitalizations in days [IQR]	234 [91–487]	485 [153–1035]	<0.001
Median length-of-hospital stay in days [IQR]	9 [4]–[15]	5 [2]–[11]	<0.001
Median number of comorbidities [IQR]	7 [4]–[9]	3 [1]–[6]	<0.001
Percentage of patients without comorbidity [95% CI]	2.8% [2.8%–2.9%]	31.1% [30.7%–31.1%]	<0.001
Percentage of in-hospital death [95% CI]	5.9% [5.8%–5.9%]	3.4% [3.3%–3.5%]	<0.001

* matching.

### COPD-patients Versus Controls

For each of the 340′948 cases a matched control (cases without COPD diagnosis) was randomly identified from the database. The controls were matched for age, gender and month of hospitalization. The characteristics of COPD cases and the controls are summarized in Table 1. Overall, patients with a diagnosis of COPD had significantly worse outcome compared to non-COPD controls. They were significantly more frequently rehospitalized (annual hospitalization rate 0.33 [IQR 0.20–0.67] vs. 0.25 [IQR 0.14–0.43] per year; 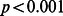), had a longer hospital stay (9 [IQR 4–15] vs. 5 [IQR 2–11] days; 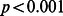), had more comorbidities (7 [IQR 4–9] vs. 3 [IQR 1–6]; 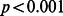), and had a significantly higher in-hospital mortality (5.9% [95% CI 5.8%–5.9%] vs. 3.4% [95% CI 3.3%–3.5%]; 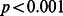).

### Comorbidities Associated with COPD

Among 8715 comorbidities, 2711 were significantly either enriched or under-represented in patients with COPD (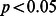). Of these, 82 with the lowest *p*-values (excluding COPD-related ICD-10 codes) were taken in the rest of the analysis (Table 2). Comorbidities associated with COPD were grouped into the following disease groups: malignant lung neoplasms (C34*); psychological disorders, tobacco/alcohol dependency (F10*, F17*, F32*, F41*); heart diseases and arterial hypertension (I10, I110, I25*, I27*, I48); other lung diseases such as pneumonia (J18*), asthma (J45*), bronchiectases (J47), respiratory failure (J96*); osteroporosis (M81*); kidney diseases (N18*); diabetes mellitus (E119); obesity, sleep apnea (E66*, G473); dependence on respirator (Z991); cachexia (R64). Few under-represented comorbidities were found, including senile cataract (H25*) and meniscus derangement (M232, M233).

**Table 2:** Prevalence (in percentage) of the most significantly over−/under-represented comorbidities in patients with COPD compared to age- and sex- matched patients without diagnosis of COPD.

ICD-10	Comorbidity	Prev.COPD	Prev.Control	Odds ratio[95% CI]	p-value
B370	Candidal stomatitis	0.88	0.21	4.30 [3.9–4.6]	<0.001
C340	Malignant neoplasm: Main bronchus	0.57	0.12	5.00 [4.4–5.5]	<0.001
C341	Malignant neoplasm: Upper lobe, bronchus or lung	2.33	0.41	5.80 [5.5–6.2]	<0.001
C343	Malignant neoplasm: Lower lobe, bronchus or lung	1.23	0.23	5.30 [5–5.8]	<0.001
C348	Malignant neoplasm: Overlapping lesion of bronchus and lung	0.55	0.11	5.10 [4.6–5.7]	<0.001
C349	Malignant neoplasm: Bronchus or lung, unspecified	1.49	0.52	2.90 [2.8–3.1]	<0.001
C771	Secondary and unspecified malignant neoplasm: Intrathoracic lymph nodes	1.11	0.27	4.10 [3.8–4.5]	<0.001
D648	Other specified anaemias	2.44	1.19	2.10 [2–2.2]	<0.001
D649	Anaemia, unspecified	3.90	2.17	1.80 [1.8–1.9]	<0.001
D751	Secondary polycythaemia	0.43	0.03	13.00 [11]–[16]	<0.001
E039	Hypothyroidism, unspecified	2.51	1.31	1.90 [1.9–2]	<0.001
E119	Non-insulin-dependent diabetes mellitus: Without complications	10.17	6.47	1.60 [1.6–1.7]	<0.001
E660	Obesity due to excess calories	2.69	1.35	2.00 [1.9–2.1]	<0.001
E662	Extreme obesity with alveolar hypoventilation	0.73	0.07	10.00 [8.9–12]	<0.001
E669	Obesity, unspecified	4.88	2.43	2.10 [2–2.1]	<0.001
E871	Hypo-osmolality and hyponatraemia	1.60	0.72	2.20 [2.1–2.4]	<0.001
F101	Mental and behavioural disorders due to use of alcohol: Harmful use	1.58	0.39	4.10 [3.9–4.4]	<0.001
F102	Mental and behavioural disorders due to use of alcohol: Dependence syndrome	5.26	1.39	3.90 [3.8–4.1]	<0.001
F171	Mental and behavioural disorders due to use of tobacco: Harmful use	12.15	1.47	9.30 [9–9.5]	<0.001
F172	Mental and behavioural disorders due to use of tobacco: Dependence syndrome	7.11	0.98	7.70 [7.4–8]	<0.001
F329	Depressive episode, unspecified	2.89	1.38	2.10 [2–2.2]	<0.001
F412	Mixed anxiety and depressive disorder	1.59	0.54	3.00 [2.8–3.1]	<0.001
G473	Sleep apnoea	3.76	1.02	3.80 [3.7–4]	<0.001
H251	Senile nuclear cataract	0.25	0.80	0.30 [0.28–0.33]	<0.001
H258	Other senile cataract	0.10	0.63	0.15 [0.13–0.17]	<0.001
H259	Senile cataract, unspecified	0.37	2.25	0.16 [0.15–0.17]	<0.001
I10	Essential (primary) hypertension	23.69	15.76	1.70 [1.6–1.7]	<0.001
I110	Hypertensive heart disease with (congestive) heart failure	4.09	1.39	3.00 [2.9–3.1]	<0.001
I119	Hypertensive heart disease without (congestive) heart failure	8.26	3.72	2.30 [2.3–2.4]	<0.001
I251	Atherosclerotic heart disease	14.11	8.99	1.70 [1.6–1.7]	<0.001
I252	Old myocardial infarction	4.37	2.53	1.80 [1.7–1.8]	<0.001
I258	Other forms of chronic ischaemic heart disease	1.65	0.78	2.10 [2–2.2]	<0.001
I259	Chronic ischaemic heart disease, unspecified	4.50	2.16	2.10 [2.1–2.2]	<0.001
I270	Primary pulmonary hypertension	2.87	0.51	5.80 [5.5–6.1]	<0.001
I272	Other secondary pulmonary hypertension	0.64	0.11	5.60 [5–6.3]	<0.001
I278	Other specified pulmonary heart diseases	1.29	0.15	8.90 [8.1–9.8]	<0.001
I279	Pulmonary heart disease, unspecified	2.02	0.13	16.00 [14]–[17]	<0.001
I420	Dilated cardiomyopathy	1.16	0.46	2.50 [2.4–2.7]	<0.001
I48	Atrial fibrillation and flutter	9.75	5.28	1.90 [1.9–2]	<0.001
I481	Persistent atrial fibrillation	2.85	1.67	1.70 [1.7–1.8]	<0.001
I500	Congestive heart failure	4.28	1.38	3.20 [3.1–3.3]	<0.001
I501	Left ventricular failure	3.83	1.55	2.50 [2.5–2.6]	<0.001
I509	Heart failure, unspecified	2.74	1.05	2.70 [2.6–2.8]	<0.001
I702	Atherosclerosis of arteries of extremities	4.65	2.15	2.20 [2.2–2.3]	<0.001
I714	Abdominal aortic aneurysm, without mention of rupture	1.57	0.62	2.60 [2.4–2.7]	<0.001
I739	Peripheral vascular disease, unspecified	2.97	1.09	2.80 [2.7–2.9]	<0.001
I872	Venous insufficiency (chronic)(peripheral)	2.23	1.07	2.10 [2–2.2]	<0.001
J13	Pneumonia due to Streptococcus pneumoniae	0.66	0.13	5.10 [4.6–5.7]	<0.001
J151	Pneumonia due to Pseudomonas	0.36	0.03	12.00 [9.6–15]	<0.001
J180	Bronchopneumonia, unspecified	1.74	0.58	3.00 [2.9–3.2]	<0.001
J181	Lobar pneumonia, unspecified	2.26	0.63	3.70 [3.5–3.8]	<0.001
J188	Other pneumonia, organism unspecified	0.96	0.30	3.30 [3–3.5]	<0.001
J189	Pneumonia, unspecified	3.19	1.08	3.00 [2.9–3.1]	<0.001
J450	Predominantly allergic asthma	0.62	0.15	4.10 [3.7–4.5]	<0.001
J451	Nonallergic asthma	0.57	0.10	6.00 [5.3–6.7]	<0.001
J47	Bronchiectasis	0.93	0.07	14.00 [12]–[16]	<0.001
J90	Pleural effusion, not elsewhere classified	1.58	0.70	2.30 [2.2–2.4]	<0.001
J960	Acute respiratory failure	4.05	0.39	11.00 [10]–[11]	<0.001
J961	Chronic respiratory failure	2.73	0.10	29.00 [26]–[32]	<0.001
J969	Respiratory failure, unspecified	2.15	0.20	11.00 [10]–[12]	<0.001
K219	Gastro-oesophageal reflux disease without oesophagitis	1.71	0.73	2.40 [2.3–2.5]	<0.001
K703	Alcoholic cirrhosis of liver	1.15	0.45	2.60 [2.4–2.7]	<0.001
M232	Derangement of meniscus due to old tear or injury	0.08	0.55	0.14 [0.12–0.16]	<0.001
M233	Other meniscus derangements	0.18	0.89	0.20 [0.18–0.22]	<0.001
M814	Drug-induced osteoporosis	0.59	0.06	9.40 [8.2–11]	<0.001
M819	Osteoporosis, unspecified	2.78	1.04	2.70 [2.6–2.8]	<0.001
N179	Acute renal failure, unspecified	1.80	0.89	2.00 [2–2.1]	<0.001
N188	Chronic kidney disease	4.39	2.13	2.10 [2–2.2]	<0.001
N189	Chronic kidney disease, unspecified	4.64	2.25	2.10 [2.1–2.2]	<0.001
N19	Unspecified kidney failure	3.03	1.67	1.80 [1.8–1.9]	<0.001
R060	Dyspnoea	1.22	0.42	3.00 [2.8–3.1]	<0.001
R64	Cachexia	1.33	0.25	5.40 [5–5.8]	<0.001
R91	Abnormal findings on diagnostic imaging of lung	1.18	0.25	4.80 [4.5–5.2]	<0.001
Y420	Glucocorticoids and synthetic analogues	0.47	0.08	6.00 [5.3–6.9]	<0.001
Z049	Examination and observation for unspecified reason	0.00	0.42	0.0034 [0.0011–0.0082]	<0.001
Z720	Tobacco use	1.03	0.14	7.30 [6.7–8.1]	<0.001
Z851	Personal history of malignant neoplasm of trachea, bronchus and lung	1.50	0.23	6.70 [6.2–7.2]	<0.001
Z864	Personal history of psychoactive substance abuse	3.17	0.65	5.00 [4.8–5.3]	<0.001
Z902	Acquired absence of lung [part of]	1.12	0.12	9.30 [8.4–10]	<0.001
Z921	Personal history of long-term (current) use of anticoagulants	3.48	2.00	1.80 [1.7–1.8]	<0.001
Z958	Presence of other cardiac and vascular implants and grafts	1.99	0.93	2.20 [2.1–2.3]	<0.001
Z991	Dependence on respirator	0.57	0.04	16.00 [13]–[20]	<0.001

The table includes the alphabetically ordered ICD-10 codes and full description, together with the odds ratios and 95% CI associated with each enriched comorbidity. The corresponding Fisher’s exact test *p*-values are given.

### Prognostic Relevance of COPD-associated Comorbidities

An increased risk of in-hospital death was found in COPD-patients with malignant lung neoplasm (C34*); pulmonary heart disease, atrial fibrillation, heart failure (I279, I48, I50*) (Wald test 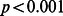). COPD-patients with comorbidities including obesity (E66*), obstructive sleep apnea (G473), osteoporosis (M819) and asthma (J45*) tended to show a relatively lower rate of in-hospital death. Comorbidities associated with a longer LOS included candidiasis (B370), anemia (D64*), depressive disorder (F329), atrial fibrillation (I48) and heart failure (I50*), asthma (J45*), respiratory failure (J96*) and cachexia (R64). COPD-patients hospitalized with comorbidities including mental disorders due to tobacco/alcohol dependency tended to have a relatively shorter LOS. The following comorbidities were associated with a shorter time to next hospitalization: neoplasm of the lung (C340 HR: 1.47 [1.33–1.64], 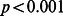; C341 HR: 1.63 [1.55–1.72], 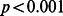; C343 HR: 1.59 [1.49–1.71], 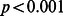; C349 HR: 1.27 [1.19–1.36], 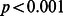); neoplasm lymphatic (C771 HR: 1.65 [1.54–1.78]); dependence on a respirator (Z991 HR: 1.86 [1.64–2.10]); pneumonectomy (Z902 HR: 1.53 [1.40–1.66]); extreme obesity with alveolar hypoventilation (E662 HR: 1.42 [1.29–1.57]); pneumonia due to pseudomonas (J151 HR: 1.42 [1.20–1.67]); secondary polycythaemia (D751 HR: 1.48 [1.34–1.64]). On the other hand, comorbidities including asthma (J450 HR: 0.44 [0.39–0.48]; J451 HR: 0.38 [0.34–0.42]) where associated with longer time to next hospitalization.

### Correlations among Comorbidities within COPD-patients

**Figure 2:**
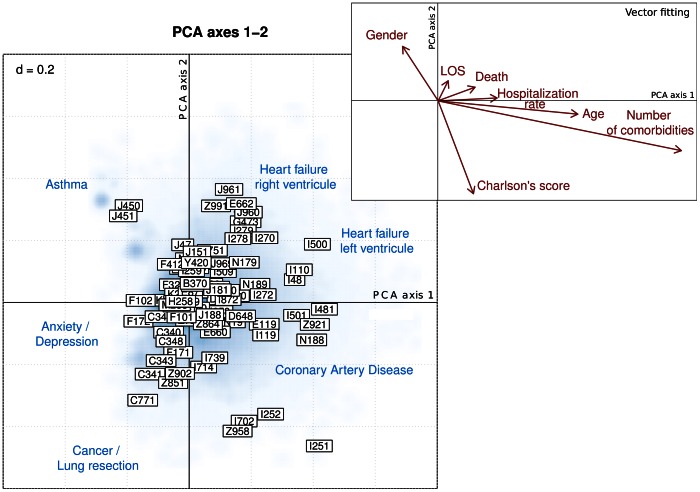
Principal component analysis biplot of comorbidities associated with COPD. Hospitalization cases (PCA scores) are represented on the first 2 principal component axes using smoothed blue colored density. Comorbidities coded according to ICD-10 are depicted by framed labels (PCA loadings). Comorbidities lying in the same directions are correlated with each other. The further away from the center of the plot, the stronger is the influence of comorbidities. External explanatory variables (including age, gender, length-of-hospital stay [LOS], in-hospital death [Death], hospitalization rate, number of comorbidities and Charlson’s comorbidity score) were fitted to the PCA and displayed in the upper right box using vectors representations (red arrows). The number displayed on the upper left corner indicates the size of the grid.

Principal component analysis (PCA) was used to explore the correlations among COPD-associated comorbidities, and to investigate the presence of comorbidity clusters within COPD patients. The biplot representation (Figure 2) summarizes the data structure of comorbidities mostly associated with COPD. A first cluster of cases lying on the upper left quadrant is characteristic of COPD-patients concomitantly diagnosed with asthma (J450, J451). The lower left part of the plot gathers comorbidities including anxiety/depression as well as mental/behavioral disorders (F101, F102, F171, F172). A third cluster of comorbidities lying on the lower part of the biplot gathers comorbidities including lung carcinomas (C341, C349, Z851) and surgical lung resections (Z902). The lower right quadrant groups together coronary artery diseases (I251, I252). A cluster of comorbidities including on the one hand heart failure of the left ventricle (I500, I110, I48), and on the other hand heart failure of the right ventricle (pulmonary heart disease I278, primary pulmonary hypertension I270, hypoventilation due to obesity E662, dependence on respirator Z991) is found on the upper right quadrant. Vector fitting procedures enable to associate external explanatory variables to the previous PCA. The strongest association (longer vector) are found with age, number of comorbidities, Charlson’s comorbidity index and gender. Age and number of comorbidities are closely correlated and are oriented toward the lower part of the PCA plot, displaying a higher mortality rate in patients having malignant lung neoplasms. Female gender was oriented towards the upper left quadrant, showing a higher proportion of females that had asthma together with COPD.

### Comorbidities in Different COPD Subtypes

**Figure 3:**
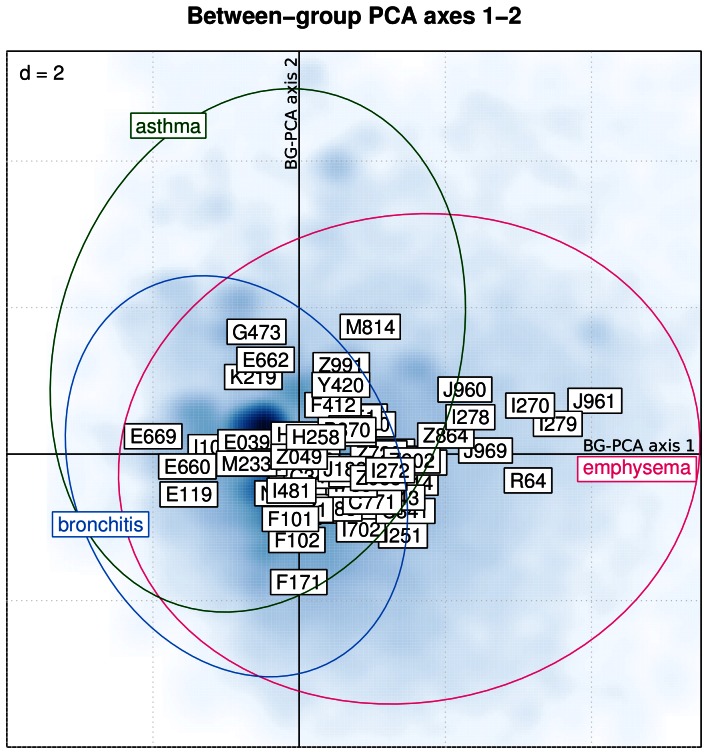
Discrimination between emphysema, bronchitis and asthma-associated COPD patients based on comorbidities using between-group principal component analysis (axes 1–2). Hospitalization cases are displayed in the first 2 axes of the between-group PCA (smoothed blue colored density). The ellipsoids summarize the dispersion of the 3 COPD subtypes in the factorial map. Comorbidities are coded according to ICD-10 codes and depicted by framed labels. The number displayed on the upper left corner indicates the size of the grid.

Supervised between-group principal component analysis was used to discriminate between subtypes of COPD, *i.e.* emphysema (J43*), chronic bronchitis (J4[0–2]*) and co-existence of COPD and asthma (other COPD patients with asthma – J4[5]–[6]* – as additional diagnosis), based on comorbidities. In this sub-analysis, only cases with strictly one of the predefined subtypes were used (*n* = 50′710). A significant discrimination (Monte-Carlo permutation test: 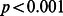) was found between the 3 subtypes (Figure 3). Table 3 provides a list of the 10 mostly associated comorbidities sorted by decreasing importance for each of the COPD subtypes. The emphysema subtype showed an association with respiratory failure (J96*), pulmonary heart disease (I27*) and cachexia (R64). Obesity (E660, E669), diabetes mellitus (E119) and arterial hypertension (I10) were more associated with the chronic bronchitis subtype. Sleep apnea (G473) and extreme obesity with alveolar hypoventilation (E662) were associated with the mixed asthma/COPD subtype.

**Table 3:** List of 10 comorbidities mostly associated with each of the 3 COPD subtypes (emphysema, bronchitis, asthma).

COPD subtypes	ICD10	Comorbidity
Emphysema		
(*n* = 17′602)		
	J961	Chronic respiratory failure
	I279	Pulmonary heart disease, unspecified
	R64	Cachexia
	I270	Primary pulmonary hypertension
	J969	Respiratory failure, unspecified
	I278	Other specified pulmonary heart diseases
	J960	Acute respiratory failure
	Z864	Personal history of psychoactive substance abuse
	J181	Lobar pneumonia, unspecified
	I714	Abdominal aortic aneurysm, without mention of rupture
Bronchitis		
(*n* = 26′615)		
	E669	Obesity, unspecified
	E119	Non-insulin-dependent diabetes mellitus: Without complications
	E660	Obesity due to excess calories
	I10	Essential (primary) hypertension
	M233	Other meniscus derangements
	E039	Hypothyroidism, unspecified
	M232	Derangement of meniscus due to old tear or injury
	F171	Mental and behavioural disorders due to use of tobacco: Harmful use
	F101	Mental and behavioural disorders due to use of alcohol: Harmful use
	F102	Mental and behavioural disorders due to use of alcohol: Dependence syndrome
Asthma		
(*n* = 6′493)		
	G473	Sleep apnoea
	K219	Gastro-oesophageal reflux disease without oesophagitis
	E662	Extreme obesity with alveolar hypoventilation
	M814	Drug-induced osteoporosis
	E669	Obesity, unspecified
	Z991	Dependence on respirator
	Y420	Glucocorticoids and synthetic analogues
	E660	Obesity due to excess calories
	F412	Mixed anxiety and depressive disorder
	I10	Essential (primary) hypertension

The lists of comorbidities are sorted in decreasing order of importance. Both ICD-10 codes and their full description are provided.

### Comparison of Patients with and without Severe COPD-exacerbation Leading to Hospitalization

Between 2002 and 2010, 71′191 hospitalizations were primarily attributed to COPD (main diagnosis) representing COPD patients with severe exacerbations leading to hospitalization. In these patients, the median length-of-hospital stay was 9 days [IQR: 5–15] which was comparable to 8 days [IQR: 4–15] in COPD patients, which never had a hospitalization primarily due to COPD (*p* NS). However, patients hospitalized due to COPD as main diagnosis at least once during the period 2002–2010 had a significantly higher number of rehospitalization (median of 2 hospitalizations [IQR 1–4] vs. 1 hospitalization [IQR 1–2]; 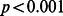).

COPD-patients without any other comorbidity also had a worse outcome compared to matched controls with a single diagnosis: the median length-of-hospital stay was 9 days [IQR 4–17] in COPD-patients vs. 2 days [IQR 1–7] in controls; increased risk of in-hospital death (OR: 2.70 [95% CI 2.42–3.02]); higher risk of re-hospitalization (OR: 4.19 [95% CI 3.92–4.48]).

## Discussion

In the current study we investigated COPD and its comorbidities in an unselected, comprehensive nation-wide population of hospitalization cases. Patients with a (co-)diagnosis of COPD had more than the double number of comorbidities, a longer length-of-hospital stay and a higher in-hospital mortality compared to matched controls. Overall, independently from the main diagnosis, which led to hospitalization in these patients, COPD seems to worsen their outcome. However, the more likely explanation of this phenomenon is that COPD could be an indicator of a complex disease with a shared pathogenic mechanism – being either anticipated aging, a systemic oxidative stress syndrome, an inflammatory disease process, or a combination of these possibilities.

Different comorbidities associated with COPD tend to group more frequently together, leading to stable disease clusters or COPD-phenotypes. A better knowledge of such phenotypes might lead to a better understanding of the shared underlying disease mechanisms. Another advantage of the knowledge of phenotypes could be that patients, who can be classified in a particular phenotype, are likely to be at risk for further not as yet diagnosed comorbidities. Such phenotyping might complement conventional COPD-phenotypes like “pink puffer” or “blue bloater”, sometimes considered as over-simplified notions.

In 1990, with an estimated 2.2 million casualties per year, COPD was identified as the sixth leading cause of death [19]. In the World Health Report of 1998, the number was estimated to 2.9 million deaths per year and COPD was classified as the fifth leading cause of worldwide mortality. Whereas other frequent causes of death are expected to decrease in the future, COPD is anticipated to gain an even larger impact on morbidity, mortality and health costs worldwide [20]–[22]. However, these alarming figures are significantly underestimated since COPD is still underdiagnosed, therefore substantially undertreated, or infrequently, sometimes inadequately treated [23]–[26]. Recent literature findings indicate that mortality in COPD is mainly caused by comorbid diseases [27]–[29]. Thus, there is a necessity to focus on COPD comorbidities and to move from traditional assessment schemes for COPD such as the well-accepted FEV_1_-based GOLD staging system to newer classification system, which also include an appraisal of the prognostic relevant systemic COPD components. In 2004, Celli and co-workers introduced the BODE-score which covers weight, subjective dyspnea level and exercise capacity as systemic attributes beside airflow obstruction [30]. The latest revision of the GOLD guidance has acknowledged this trend and distanced itself from its former solely FEV_1_-based staging system. Instead, the new GOLD report recommends a combined COPD assessment comprising symptoms, airflow obstruction, rate of exacerbations, and comorbidities [31].

Limitation of the study include the fact that only hospitalization cases could be identified. Most COPD exacerbations are treated in an outpatient setting. Thus, conclusions are mostly driven by more advanced COPD disease stages and more sever exacerbations necessitating hospitalization. It is likely that COPD was only coded as a diagnosis in case of a relevant and active form of disease. The coded diagnoses rely on the coding skills of the clinician and bias can occur since no other sources of confirmation (such as spirometry) are available. Because COPD is likely to be an underdiagnosed disease, some of our controls might correspond to patients with undiagnosed COPD. Inversely, this study deals with diagnosed comorbidities, hence COPD subjects potentially have a higher likelihood of being diagnosed with other diseases. Due to the nature of the database we could not investigate survival, but in-hospital mortality instead. Data on smoking history was not available from the database of the Swiss Federal Office for Statistics, and, thus, we could not adjust for this important potential confounding factor in our analyses. On the other hand, the studied database provides comprehensive nation-wide coverage resulting in a relevant number of cases over time. In this large database, all patients hospitalized in any Swiss hospital were registered with specific informations including demographic, geographic and clinical parameters as well as their diagnoses established at hospital discharge. Therefore, this database provides a unique source of information with regard to this study.

In conclusion, COPD is an undesirable condition because of its association with the presence of twice as many comorbidities and worse overall outcome compared to matched controls. When treating patients with COPD, a special focus on early detection and treatment of comorbidities might improve overall outcome.

## Materials and Methods

### Hospitalization Database

All our calculations are derived from a dataset provided by the Swiss Federal Office for Statistics. These data were imported into a relational SQL database (MySQL, version 5.5.24). The database was interfaced with the R statistical software (version 2.15.0) [32], using the dedicated package RMySQL. The database includes 12′888′075 entries (5′633′564 unique patients) corresponding to all hospitalizations in Switzerland between 2002 and 2010. Each patient in this database is identified uniquely so that it is possible to track the different rehospitalizations of every patient. The database includes geographical information (patient residence location, and Swiss canton where the patient was hospitalized). Temporal information is also available (year and month when the patient was hospitalized). Other information included length-of-hospital stay, age at admission and reason/type of discharge (including death).

The patients’ diagnosis list included one main diagnosis as well as up to 50 additional diagnoses coded using the International Classification of Disease version 10 (ICD-10) codes: http://www.who.int/classifications/icd/en/.

### COPD Definition and Case-control Study Design

For the current study, COPD was defined by the following ICD-10:

- J40: bronchitis, not specified as acute or chronic
- J41: simple and mucopurulent chronic bronchitis
- J42: unspecified chronic bronchitis
- J43: emphysema
- J44: other chronic obstructive pulmonary disease

Our case population was defined as any hospital case with the diagnosis of COPD either as main or concomitant diagnosis. Only elderly patients were included in the study (age at hospitalization 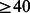 years). Geriatric, obstetric, psychiatric hospitalization and cases of rehabilitation were excluded from our study. Cases with COPD as main diagnosis were considered as “acute COPD” cases. Cases with COPD as co-diagnosis were considered as “chronic COPD” cases. A control population (no COPD diagnosis) was built matching the case population for age, gender, and month of hospitalization.

### Comorbidities

COPD-comorbidities were defined as any additional diagnosis coded by the ICD-10 nomenclature. The Charlson’s comorbidity index [33] was used to assign a predicted mortality score to patients.

### Statistical Analysis

Descriptive statistics is reported as median and inter-quartile-range (IQR). Fisher’s exact test was used to test the enrichment of COPD-associated comorbidities. Time-to-event data were analyzed using Kaplan-Meier estimates and Cox-proportional hazards regression. Results are reported as hazard ratio (HR) with 95% confidence intervals (CI) together with the Wald test statistic. Logistic regression with generalized estimating equations (using working independence correlation structure) was used to test associations in our case-control design with one or several covariates while accounting for within-patient variability. Results are reported as odds-ratio (OR) with 95% CI together with the Wald test statistic.

Principal component analysis (PCA) was used to explore how comorbidities are related to each other and how hospitalized patients are grouped according to comorbidities. The main table of interest is the matrix summarizing the presence/absence of comorbidities in COPD patients. Scaled PCA was applied to the comorbidity table. The role played by external explanatory variables, including age, length-of-hospital stay, gender, in-hospital death, hospitalization rate, Charlson’s comorbidity index and number of comorbidities, was further explored using vector fitting procedure to identify the directions of maximal correlation with the external variables in the PCA space [34]. PCA results were directly displayed using biplot representation [35]. PCA biplots are organized as follows: due to the very large number of hospitalization cases (rows of the comorbidity table), smoothed color density image was used to depict the density of cases in the biplot representation (PCA scores); comorbidities (columns of the comorbidity table) are represented using framed labels (PCA loadings); external explanatory variables are superimposed to the biplot using vector representations pointing to the direction of most rapid change and whose length is proportional to the correlation between the PCA configuration and the explanatory variable.

In order to explore how pre-defined COPD phenotypes are influenced by comorbidity distribution, between-group principal component analysis was used. Between-group PCA is a supervised counterpart of PCA used when an additional categorical variable is introduced as a constrain in the PCA model [36], [37]. Between-group PCA finds combinations of variables that best discriminate between pre-defined groups by maximizing the ratio of between- over within-group inertia. Between-group PCA scatter plots are organized as follows: hospitalization cases are represented using smoothed color density image (between-group PCA scores); comorbidities are displayed using framed labels (between-group PCA loadings); the spread of cases within each COPD phenotypes is summarized using inertia ellipses. All statistical computations were done using the R statistical software (version 2.15.0) [32]. The following extension packages were used: survival, geepack, ade4 and vegan.
